# Maximal left ventricular wall thickness: a comparison between CMR and echocardiography in hypertrophic cardiomyopathy

**DOI:** 10.1186/1532-429X-15-S1-P169

**Published:** 2013-01-30

**Authors:** Celia P Corona-Villalobos, Lars Sorensen, Linda Chu, Theodore Abraham, Ihab R Kamel, Stefan L Zimmerman

**Affiliations:** 1 Radiology and Radiological Science, Johns Hopkins Hospital, Baltimore, MD, USA; 2 Department of Cardiology, Johns Hopkins Hospital, Baltimore, MD, USA

## Background

Echocardiography (ECHO) is used for diagnosis and management of hypertrophic cardiomyopathy (HCM). Morphologic diagnosis is based on the presence of myocardial thickness of >15mm in adults or a septum to posterior wall ratio of >1.5. Septal wall thickness (WT) >30 mm is one of several high risk features that are used to guide the decision for prophylactic implantable cardioverter-defibrillator (ICD) placement. Cardiac magnetic resonance imaging (CMR) offers improved contrast resolution over ECHO and precisely defines myocardial WT in HCM.CMR is used in suspected HCM when ECHO is inconclusive for diagnosis or when additional information is needed.

**Figure 1:**
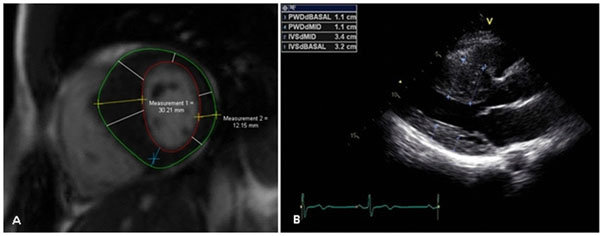
Maximal wall thickness measures in end diastolic phase by a) CMR and by b) ECHO at the basilar septum and posterior wall.

We tested the hypothesis that there are differences in maximal left ventricular myocardial WT as measured by CMR and ECHO in subjects with HCM due to asymmetric septal hypertrophy (ASH).

## Methods

64 subjects with HCM and ASH pattern (mean age 51 years, range 18-88 years), underwent ECHO and CMR within the same day. Maximal WT by ECHO was measured on parasternal long axis view in the anteroseptal and the basilar posterior wall. WT by CMR was measured on the short axis view manually to obtain maximal dimensions in the same segments as those obtained by ECHO.

## Results

Overall, mean maximal septal WT for all patients was less by CMR compared with ECHO, but not statistically significant (19.1+4.7 and 20.5+4.4, respectively, p>.05). We stratified our population into two groups by maximal septal WT: A) >15-19.9mm and B) >20mm. CMR showed less mean maximal septal WT than ECHO, which was significant only in group B [group A (16.8+3.6 and 17.1+1.5, respectively, p>.05) and group B (21.8+4.4 and 24.7+3.1, respectively, p<.05)].The posterior wall showed greater WT for CMR compared with ECHO, which was significant only in group B [group A (12.3+2.7 and 11.1+2.7, p>.05) and B (13.7+3.8 and 11.8+2.6, respectively, p<.05)]. On a per patient basis, the difference between ECHO and CMR for maximal septal WT was greatest in group B but significant (p<0.05) for both groups (group A -0.3+3.6mm and group B -2.9+3.9mm). On per patient basis, the difference between ECHO and CMR for the posterior wall was greater but significant only in group B (group A 1.2+3.1, p>.05 and 1.8+4.2mm, p<.05). The septal to posterior wall ratio calculated by CMR and ECHO was also significantly different (Group A 1.4+0.4 and 1.6+0.4 and Group B 1.7+2.2 and 2.2+0.5 respectively, p<.05). ECHO and CMR showed good correlation for the septal WT (r=.62, p<.001); however there was a poor correlation for the posterior wall (r=.36, p<.001).

## Conclusions

In subjects with WT >20 mm echocardiography shows systematically higher septal WT and lower posterior WT resulting in higher septal to posterior wall ratio compared to CMR. These findings have potential clinical implications for diagnosis and management of subjects with HCM.

## Funding

No funding was given for this project.

**Table 1:** Demographics and myocardial wall thickness in our cohort stratified into two groups

	Wall thickness 15-19.9 mm (n=35)	Wall thickness ≥20 mm (n=29)
Demographics Age (years) BSA (m2) Gender Males Female	47.1+15.7 2.1+0.4 23 (66%) 12 (34%)	53.7+15.4 2.1+0.3 22 (76%) 7 (24%)

Maximal Septal Wall Thickness (mm) MRI Echo Per patient difference (MRI - Echo)	16.8+3.6 17.1+1.5 -0.3+3.6	21.8+4.4 24.7+3.1 -2.9+3.9

Maximal Posterior Wall Thickness (mm) MRI Echo Per patient difference (MRI - Echo)	12.3+2.7 11.1+2.7 1.2+3.1	13.7+3.8 11.8+2.6 1.8+4.2

Septum to Posterior Wall Ratio MRI Echo Per patient difference (MRI - Echo)	1.4+0.4 1.6+0.4 -0.2+0.6	1.7+0.6 2.2+0.5 -0.5+0.6

Values are reported as mean + standard deviation unless state otherwise.

